# Recent Advances and Perspective of Studies on Phlegm Syndrome in Chinese Medicine

**DOI:** 10.1155/2016/6463270

**Published:** 2016-02-14

**Authors:** Zhiguo Zhang, Jingqing Hu

**Affiliations:** Institute of Basic Theory, China Academy of Chinese Medical Sciences, No. 16 Nanxiao Road, Dongzhimennei, Beijing 100700, China

## Abstract

This review paper summarized the current situation of studies on the essence of phlegm syndrome and relation between phlegm syndrome, diseases, and therapeutics based on published English articles. In studies on the essence of phlegm syndrome, omic technologies were used to explore the molecular basis of phlegm syndrome; in studies on relation between phlegm syndrome and diseases, discovery of markers of phlegm syndrome in diseases becomes a hotspot; the distribution of phlegm syndromes in some common chronic diseases was found; in the therapy of phlegm syndrome, two therapeutic models, treatment with CM formula and treatment with a combination of CM formula and Western medicine, were used most frequently. It is certainly that using one omic technology is not able to deal with the complexity of phlegm syndrome and that the use of a combination of multiple omic methods will be a trend in future studies. Meanwhile, for rapidly increasing clinical research quality of phlegm syndrome, a series of agreed criteria, such as syndrome diagnostic criteria and efficacy criteria clinical studies of phlegm syndrome, needed to be established urgently, and there was an urgent need of standardizing syndrome names in English.

## 1. Introduction

Phlegm is one of endogenous pathological factors in Chinese medicine (CM). Phlegm begins to appear in the body when the body fluid is not transported by vital energy normally and accumulates in certain parts of the body after being condensed. As phlegm can be retained in Zang- and Fu-viscera and can flow randomly in various directions into meridians and collaterals, phlegm syndrome appears in almost all diseases [1]. Common symptoms phlegm could cause include expectoration, chest tightness, gastrectasia, anorexia, nausea, vomiting, dizziness, and body mass. In addition, thick tongue coating and slippery pulse are usually considered to be typical diagnostic signs.

A number of English articles have been published on phlegm syndrome in the past decades, but review papers outlining the research status of phlegm syndrome are still lacking. This review paper, based on published English articles, summarized the current situation of studies on the essence of phlegm syndrome and relation between phlegm syndrome, diseases, and therapeutics.

## 2. Studies on the Essence of Phlegm Syndrome

Studying the essence of a syndrome has become a key challenge in the field of studies on CM. Though the omic technologies could provide integral, systemic, and dynamic technology platforms for studies of syndrome, the progress toward understanding the essence of syndrome was still slow. Three omic methods, metabolomics, genomics, and proteomics, were used in studies for discovering the essence of phlegm syndrome.

### 2.1. Metabolomics

Metabolomics encompasses the dynamics, composition, and analysis of metabolites, enabling the observation of changes in the metabolic network of the human body associated with disease. Being from the point of view of the whole organism, metabolomics provides an opportunity to study the essence of phlegm syndrome to an unprecedented level.

In young adults with essential hypertension, patients with phlegm-heat syndrome had a small metabolite disorder in the serum compared with the healthy control group. In phlegm-heat syndrome patients, there was an increase in seven metabolites [low-density lipoprotein/very low-density lipoprotein, N-acetyl glycoprotein, O-acetyl glycoprotein, lipids -CH_2_-CH_2_COa, lipids -CH_2_-CH=a, lipids -CH=COa, and lipids -CH=CHa] and decrease in 10 metabolites (high-density lipoprotein, phosphatidylcholine, lactate, alanine, choline/phosphocholine, phosphorylcholine, glucose, 3-hydroxybutyrate, citrate, and glutamine) [2].

In coronary heart disease (CHD), Zhao et al. found 20 plasma differential metabolites for discriminating phlegm syndrome from blood stasis syndrome. Of the 20 differential metabolites, there was an increase in 11 metabolites (L-leucine, uric acid, L-phenylalanine, L-tryptophan, ubiquinone, 17,20-dimethyl prostaglandin, isoquinoline N-oxide, PGH2-EA, stearamide, deoxycholic acid 3-glucuronide, and octadecyl fumarate) and decrease in 9 metabolites (ubiquinone, N-palmitoyl taurine, N-oleoyl taurine, deoxycholic acid disulfate, L-tyrosine, uridine, itaconic acid, deoxyuridine monophosphate, and eicosapentaenoic acid ethyl ester) in the plasma of CHD patients [3].

Batur et al. found that compared with the control group neoplasm patients with phlegm-stasis syndrome had low plasma concentrations of leucine, isoleucine, valine, alanine, tyrosine, histidine, citrulline, glycoprotein, glutamine, myoinositol, scyllo-inositol, creatine, *α*-glucose, *β*-glucose, and lactate and high plasma concentrations of very low-density lipoprotein, low-density lipoprotein, unsaturated lipid, formate, acetone, acetate, acetoacetate, pyruvate, *β*-hydroxybutyrate, carnitine, and malonic acid [4].

Results from the metabolomics analysis of Cha et al. demonstrated that two plasma metabolites were identified as lysophosphatidylcholines (LPCs), LPC 18:2, and LPC 20:3, having an unsaturated acyl chain and showed lower levels in the phlegm syndrome than in the nonphlegm syndrome of acute cerebral infarction patients, suggesting that the plasma LPCs with polyunsaturated fatty acid groups were associated with phlegm syndrome and that the variation of plasma lipid profiles may serve as a potential biomarker for the diagnosis of phlegm syndrome [5].

A study conducted by Bai and Song showed that five plasma metabolites had significant differences between hyperlipidemia and atherosclerosis patients with phlegm-blood stasis syndrome and those without phlegm and blood stasis syndromes. These were identified as urine, isoleucine, glucuronic acid, palmitic acid, and glycerol. The concentrations of four metabolites in the plasma of patients with syndrome of phlegm and blood stasis were higher than those with syndromes without phlegm and blood stasis, whereas the glycerol concentration was lower [6].

The differential metabolites associated with phlegm syndrome are listed in Table 1.

### 2.2. Genomics

Researchers from Korea explored the relation between the polymorphism of specific genes and phlegm syndrome.

Based on the analysis of gene polymorphism of peripheral blood, Lim et al. found that A-3826G and A-1766G uncoupling protein-1 polymorphisms, which are related to obesity, might be candidate genetic markers for the dampness-phlegm syndrome in patients with stroke [7]. Additionally, Ko et al. found that neuropeptide Y (NPY) gene polymorphism was associated with dampness-phlegm syndrome in stroke patients. Among the five single-nucleotide polymorphisms (G-1484A, C-1471T, C-399T, A1201G, and C5325T) of NPY, the T allele of C-399T had a negative association with the dampness-phlegm pattern by decreased levels of serum cholesterol, which were significantly higher in dampness-phlegm pattern patients compared with non-dampness-phlegm pattern patients [8].

### 2.3. Proteomics

It is in only one study that the proteomic method was used for exploring the essence of phlegm syndrome [9]. In this study, the differentiated proteins between the phlegm syndrome group and the control group were regarded as the material foundation of phlegm syndrome. Researchers found that the best biomarkers of abundant phlegm-dampness syndrome in essential hypertension patients were protein peaks with the mass-to-charge ratio (*m*/*z*) of 9334.958 *m*/*z* (the expression increased), 9280.191 *m*/*z* (the expression decreased), 8030.794 *m*/*z* (the expression increased), and 2941.551 *m*/*z* (the expression increased).

## 3. Relation between Phlegm Syndrome and Diseases

The syndrome is a specific concept in CM. The syndrome summarizes and includes symptoms, signs, causes, locations, properties, and tendency of disease during a period. In CM, a disease can present different syndromes in different patients. Therefore, identifying syndromes is the crucial link in the procedure of CM diagnosis and treatment.

### 3.1. Distribution of Phlegm Syndrome in Diseases

Theoretically, phlegm syndrome can present in almost all diseases. In the past decades, a line of studies proved that the syndrome associated with phlegm was one of the main syndromes in some diseases.

#### 3.1.1. Coronary Heart Disease

Results from a study conducted by Wang et al. showed that Xin (heart) phlegm with turbid fluid (38.0%) was ranked fourth in occurrence frequency among the eight main syndrome elements of CHD [10]. Lately, Ren et al. found that phlegm syndrome was one of the most common syndromes in CHD patients and that the proportions of patients with phlegm syndrome (68.9%) were generally higher in the south group [11].

#### 3.1.2. Ischemic Stroke

Cheng et al. reported that the most frequent CM syndromes associated with an acute ischemic stroke were wind syndrome, phlegm syndrome, and blood stasis syndrome [12]. Similarly, Liu et al. found that phlegm and blood stasis syndromes exist through the whole process of this disease and hold that inflammation and apoptosis induced by cerebral vascular injury in the pathological processes of an ischemic stroke are more prominent in the excess syndrome state like phlegm-dampness and blood stasis [13].

#### 3.1.3. Other Diseases

A retrospective study based on literature analysis and conducted by Wang et al. showed that the ratio of phlegm syndrome was 19.03% in 13,272 patients with essential hypertension and ranked twice in two-factor syndromes [14]. Bao et al. observed the distribution of syndromes of patients with nasopharyngeal carcinoma and found that phlegm syndrome accounted for the second highest proportion at 32.7% (48/147). In addition, the patients with phlegm or blood stasis-phlegm syndrome had a higher incidence of cervical lymph node metastases compared with patients with other syndromes (*P* < 0.05) [15].

### 3.2. Markers of Phlegm Syndrome

Syndrome differentiation is mainly based on the information collected by four diagnostic methods of CM, namely, observation diagnosis, auscultation and olfaction diagnosis, inquiry diagnosis, and pulse diagnosis. It takes a very long time for practitioners of CM to be proficient in four diagnostic methods. For practitioners of non-CM, four diagnostic methods are elusive and hard to learn. Therefore, some researchers strive to discover the markers of syndrome as an alternative of four diagnostic methods.

Wang et al. found that the levels of three indices (Von Willebrand factor, D-dimer, and fibrinogen) increased in CHD patients and were higher in phlegm syndrome patients than in non-phlegm syndrome patients (*P* < 0.05). They thought these three markers could be taken as the markers for differentiating phlegm syndrome in CHD patients [16]. Sun et al. discovered a significant decrease in E-cad/intercellular adhesion molecule-1 expression in phlegm syndrome patients with gastric carcinoma compared to those with the deficiency in both qi and blood and the deficiency-cold of stomach and spleen syndromes [17].

Some nonbiochemical indices were also considered as markers of phlegm syndrome. A study on correlation between cognitive functions and syndromes in amnestic mild cognitive impairment (aMCI) showed that instant story recall and delayed story recall scores of patients were, respectively, correlated with turbid phlegm blocking syndrome (*r* = −0.11, *r* = −0.27; *P* = 0.021, *P* = 0.000) [18].

## 4. Therapy of Phlegm Syndrome

Phlegm syndrome is one of most common syndromes in the clinical practice of CM. Because phlegm syndrome forms via different pathological pathways, so many CM formulas for treating phlegm syndrome exist in Chinese medical classics. In clinical reports associated with the treatment of phlegm syndrome, single CM formula and a combination of CM formula and Western medicine were two main therapeutic models.

### 4.1. Treatment with Chinese Medicine Formula for Resolving Phlegm

Twelve-week treatment with Bushenhuatanyizhi granules, a CM formula for reinforcing kidney-essence, removing phlegm, and promoting mental therapy, was able to increase mini mental state examination scores and decrease Activity of Daily Living Scale scores of patients with Alzheimer's disease compared with those before treatment [19]. In a study on early hypertension, Yinian Jiangya Yin, a CM formula for dissolving phlegm-stasis, was proved to have a more potent effect in relieving symptoms, lowering blood pressure, and regulating imbalanced condition between endothelin and nitrogen monoxide in serum compared with Tianma Gouteng Yin [20]. Results from a 12-week randomized, double-blind, and parallel-controlled trial for evaluating the clinical efficacy in the treatment of amnestic mild cognitive impairment suggested that the CM decoction for tonifying the kidney and resolving phlegm and blood stasis could improve global cognition, attention function, and some clinical symptoms in patients with aMCI [21]. Yang et al. found that the antihyperlipidemic effect of Danshen Jueming granules (a CM for replenishing qi, promoting blood circulation, and resolving phlegm) was obviously superior to Xuezhikang capsules (a commonly used antihyperlipidemic drug) in reducing plasma total cholesterol and low-density lipoprotein cholesterol as well as in reducing the ratio of thromboxane B2 to 6-keto-prostaglandin F1*α*, D-dimer, and fibrinogen after 1-month treatment [22].

### 4.2. Treatment with a Combination of Chinese Medicine Formula for Resolving Phlegm and Western Medicine

In a multicenter, randomized, double-blind, placebo-controlled clinical trial on AECOPD patients with the syndrome type of phlegm-heat obstructing the lungs, treatment with a combination of Xuan Bai Cheng Qi granules (a CM formula for clearing heat and removing phlegm in Fei) and routine pharmacotherapy showed a better efficacy in improving symptom scores, FEV1, FVC, FEV1%, PaO_2_, and PaCO_2_ compared with conventional medicine and placebo [23]. In addition, a combination of Tanreqing injection and routine pharmacotherapy was also proved to have a stronger effect on improving Chinese medical signs and symptoms in patients with AECOPD compared with routine pharmacotherapy only [24].

Besides AECOPD, treatment with a combination of CM formula and Western medicine showed potent efficacy on unstable angina pectoris (UAP). Liu et al. found treatment with a combination of Quyu Xiaoban capsules (a CM formula for dissolving phlegm-stasis) and aspirin can decrease plasma platelet aggregation rate and P-selectin level to a greater extent than treatment with aspirin alone, in addition to attenuating anginal attacks and improving CM symptoms and signs in patients with UAP and phlegm and blood stasis syndrome [25].

In animal experiment, Wang et al. reported that the use of a combination of WRCP (a warming and relieving cold phlegm formula) and 5-fluorouracil was more effective in inhibiting tumor growth than either agent alone and may have potentially additional benefit in improving the general condition and immunity of the model mice compared with a single use [26].

## 5. Conclusion and Prospective

For understanding CM syndromes, it is very important to discover the biological essence of syndromes. Omic technologies and bioinformatics methods were suitable for studies of CM syndromes [27]. Less than 20 articles were retrieved reporting the essence of phlegm syndrome. In only one study using proteomics, the researchers merely determined the mass spectral range of differentially expressed proteins and did not identify these proteins furthermore. In contrast, the metabolomic method was used more frequently in this kind of study considering its sensitivity. A number of differential metabolites between patients with phlegm syndrome and healthy control group were discovered (Table 1). From these differential metabolites, it was found in this review that phlegm syndrome in different diseases was closely associated with increasing lipid metabolites, but there was a big difference in these metabolites between different diseases with phlegm syndrome. Until now, none of metabolites has been recognized as a metabolic marker for phlegm syndrome. As a future work, the researchers are encouraged to explore the underlying biological essence of phlegm syndrome by the use of a combination of various omic methods like proteomics, metabolomics, genomics, metagenomics, and so forth.

Rather than using omic methods, more researchers explored the relation between phlegm syndrome and biochemical indices or nonbiochemical indices, looking forward to find potential markers of phlegm syndrome. However, before these potential markers were accepted by clinical medical practitioners, clinical trials for validating the specificity were necessary.

Phlegm syndrome was a common syndrome in many diseases, especially in CHD, stroke, and cancers, but pure phlegm syndrome was rare in these diseases and phlegm syndrome was usually mixed with other syndromes, such as phlegm-heat syndrome and phlegm-stasis syndrome. Phlegm syndrome was found to have several synonyms in many papers. For instance, phlegm syndrome could be named “phlegm congealment type” [15], “phlegm-dampness syndrome” [13], or “turbidity-phlegm blocking syndrome” [16] in different studies. These unstandardized syndrome names hindered the understanding implication of phlegm syndrome to some extent. With the emergence of more and more studies associated with the syndrome of CM conducted by researchers coming from countries other than China, there was an urgent need of standardizing syndrome names in English.

In most of clinical studies on phlegm syndrome, the aim of the study was to observe the syndrome distribution of a disease or to assess the efficacy of CM formulas for dissolving phlegm. It is recognized that syndrome diagnostic criteria or efficacy criteria played a key role in maintaining result reliability when conducting a study on observing syndrome distribution or assessing the efficacy of CM. However, in many clinical studies on phlegm syndrome, investigators used different diagnostic criteria or efficacy criteria in clinical studies on phlegm syndrome, even if the disease they researched was the same. For example, Wang et al. used the “Guiding Principle of Clinical Research on New Drugs of Traditional Chinese Medicine (2002)” as syndrome diagnostic criteria of CHD [16], while syndrome diagnostic criteria of CHD in the study of Ren et al. were based on “criteria for TCM syndrome differentiation of patients with CHD (revised in 1990)” released by the China Society of Integrated Traditional Chinese and Western Medicine [11]. Obviously, if researchers wanted to increase the research quality rapidly, a series of agreed criteria, such as syndrome diagnostic criteria and efficacy criteria clinical studies of phlegm syndrome, needed to be established urgently.

## Figures and Tables

**Table 1:** Differential metabolites associated with phlegm syndrome.

Comparisons	Sample	Increased differential metabolites	Decreased differential metabolites
Essential hypertension patients with phlegm-heat syndrome versus healthy control [2]	Serum	Low-density lipoprotein Very low-density lipoprotein N-Acetyl glycoprotein O-Acetyl glycoprotein Lipids	High-density lipoprotein Phosphatidylcholine Lactate Alanine Choline/phosphocholine Phosphorylcholine Glucose 3-Hydroxybutyrate Citrate Glutamine

Coronary heart disease patients with phlegm from blood stasis syndrome versus healthy control [3]	Plasma	L-Leucine Uric acid L-Phenylalanine L-Tryptophan Ubiquinone 17,20-Dimethyl prostaglandin Isoquinoline N-oxide PGH2-EA Stearamide Deoxycholic acid 3-glucuronide Octadecyl fumarate	Ubiquinone N-Palmitoyl taurine N-Oleoyl taurine Deoxycholic acid disulfate L-Tyrosine Uridine Itaconic acid Deoxyuridine monophosphate Eicosapentaenoic acid ethyl ester

Neoplasm patients with phlegm-stasis syndrome versus healthy control [4]	Plasma	Very low-density lipoprotein Low-density lipoprotein Unsaturated lipid Formate Acetone Acetate Acetoacetate Pyruvate *β*-Hydroxybutyrate Carnitine Malonic acid	Leucine Isoleucine Valine Alanine Tyrosine Histidine Citrulline Glycoprotein Glutamine Myoinositol Scyllo-inositol Creatine *α*-Glucose *β*-Glucose and lactate

Acute cerebral infarction patients with dampness-phlegm syndrome versus non-dampness-phlegm syndrome [5]	Plasma		Lysophosphatidylcholines (18:2) Lysophosphatidylcholines (20:3)

Hyperlipidemia and atherosclerosis patients with phlegm-blood stasis syndrome versus non-phlegm-blood stasis syndromes [6]	Plasma	Urine Isoleucine Glucuronic acid Palmitic acid	Glycerol
